# Supplementary material for: The association between nurses’ physical activity counselling and patients’ perceptions of care quality in a primary care facility in Ghana
Source: PLoS One. 2022 Jul 21;17(7):e0270208. doi: 10.1371/journal.pone.0270208 (PMC9302826; doi:10.1371/journal.pone.0270208)
Supplement: S1 Appendix — (DOC) [file pone.0270208.s001.doc]

SUPPLEMENTAL DIGITAL CONTENT 1

Appendix A. Items and dimensions of NPAC

| Domain | Code | Item |
| --- | --- | --- |
| PA recommendation | NP1 | Indicate to you that exercise is a healthy habit |
|  | NP2 | Tell you how much time you should spend on exercise per day or week |
|  | NP3 | Tell you what you should do to avoid the side effects of excessive physical activity or high-intensity exercise |
|  | NP4 | Ask you to measure or record your routine exercise levels using devices such as your mobile phone and pedometer |
| Follow-up | NP5 | Follow up with you to assess your progress in routine exercising |
|  | NP6 | Follow up with you to set goals for routine physical activity |
|  | NP7 | Follow up with you to solve problems from exercising e.g. body pains, dislocations |
