# Supplementary material for: The association between nurses’ physical activity counselling and patients’ perceptions of care quality in a primary care facility in Ghana
Source: PLoS One. 2022 Jul 21;17(7):e0270208. doi: 10.1371/journal.pone.0270208 (PMC9302826; doi:10.1371/journal.pone.0270208)
Supplement: S2 Appendix — (DOC) [file pone.0270208.s002.doc]

Appendix B. Items and dimensions of care quality indicators

| Construct | Code | Item |
| --- | --- | --- |
| Care quality | CQ1 | Polite attitudes of employees |
|  | CQ2 | Employees explaining the details to you in health care |
|  | CQ3 | Employees listening attentively to you |
|  | CQ4 | Employees understanding and considering your situation |
|  | CQ5 | The worker's sense of closeness and friendliness to you |
|  | CQ6 | The hospital knowing your needs |
|  | CQ7 | Hospital understanding your problems |
|  | CQ8 | Degree of securing advanced medical equipment by hospital |
|  | CQ9 | Degree of securing medical staff with advanced skills and knowledge by the hospital |
|  | CQ10 | Degree of convenience of the facilities |
|  | CQ11 | Degree of cleanliness of employee uniforms |
|  | CQ12 | Overall cleanliness of the hospital |
|  | CQ13 | Degree of efforts for providing a comfortable and safe environment for patients |
|  | CQ14 | Degree of the feeling that doctors would not make misdiagnoses |
|  | CQ15 | Degree of the feeling that nurses would not make mistakes |
|  | CQ16 | Degree of confidence about the medical proficiency of this hospital |
|  | CQ17 | Attitudes about not using unnecessary medication |
|  | CQ18 | Degree of efforts for proving appropriate treatment methods |
|  | CQ19 | Reasonable medical expenses |
|  | CQ20 | Appropriateness of cost for medical service provided |
|  | CQ21 | Appropriateness of care service provided |
|  | CQ22 | Recognition and efforts for the best treatment by the medical staff |
|  | CQ23 | Improvement in your medical condition as a result of efforts and treatment by the medical staff |
|  | CQ24 | Degree to which your medical condition has improved after using this hospital care |
|  | CQ25 | Degree to which employees give you explanations to prevent related diseases |
|  | CQ26 | Degree of efforts and willingness by the hospital to prevent you from contracting diseases |
| Patient satisfaction | PS1 | I am satisfied with the efforts this hospital/clinic makes towards patients |
|  | PS2 | I am satisfied with the relationship I have with this hospital/clinic |
| Patient loyalty | PL1 | I would always visit this hospital for medication and treatment |
|  | PL2 | I would recommend this hospital to my friends |
|  | PL3 | I am ready to travel extra mile to reach this hospital |
