# Supplementary material for: The association between nurses’ physical activity counselling and patients’ perceptions of care quality in a primary care facility in Ghana
Source: PLoS One. 2022 Jul 21;17(7):e0270208. doi: 10.1371/journal.pone.0270208 (PMC9302826; doi:10.1371/journal.pone.0270208)
Supplement: Attachment — Submitted filename: Response to Reviewers and Editors.doc [file pone.0270208.s005.doc]

**Response to the Editors and Reviewers**

We are indeed grateful to the editors and reviewers for their expert comments. Despite disruptions from COVID-19, you were able to provide timely feedback. Above all, the comments are extremely helpful; we have incorporated all of them into our revision. Our responses to the comments are highlighted for your easy tracking.

**Additional Editor Comments:**

**Comment**: On page 10 of the manuscript, the authors talk about "customer satisfaction". It would be more appropriate to say "patient satisfaction". It is true that the customer in the healthcare system is the patient. However, it is more accurate to speak directly of the patient.

**Our response**: We agree with you completely. We have changed that wording.

**Comment**: The authors sometimes use "counselling" and sometimes "counselling". Please decide on one form and always use it.

**Our response**: This is true. We have used ‘counselling’ throughout the manuscript.

**Comment**: It would be useful to make a graph showing the relationships between the variables, so that the reader can better follow the text. You can see graphs of this type in many articles. You can see what I mean in the following article (it is not necessary to use it as a bibliographical reference): <https://doi.org/10.3390/ijerph18052304>

**Our response**: We are very grateful for this. We thought as much. Figure 1 has been added as the structural model with all the relationships tested. By providing the structural (hypothetical) model, we are able to depict the role of the potential confounding variables, including those removed in the sensitivity analysis, in the study. Thanks once again for this.

**Reviewer #1**

**Comment:** I am delighted to review this paper. The study is important for the patients in fast recovery, especially post-operative patients related to bone fracture, nerve injury, delivery etc, by decreasing the burden of disease and health care costs as a result increasing productivity and development.

Despite the good nature of the paper, here are my concerns below.

**Our response**: Your comments are highly relevant. Thanks

**Comment**: 1. Study population: your study population was patients in the OPD, is that applicable to practice PA counseling for all patients came OPD? I think PA counselling for patients having specific diseases like hypertension, DM etc. Authors study population should be clear

**Our response**: Thanks for this. We have provided details in the manuscript under methods (selection). Only outpatients met the inclusion criteria as in-patients were isolated and did not have the opportunity to practice PA counselling. Other reasons can be found in the manuscript.

**Comment**: 2. Method:” Covariates with p>0.25 were removed and those with p≤0.25 were kept for the second level of the sensitivity analysis” no need to write in this way it is better to write Covariates with p≤0.25 were included for the multivariate analysis (second analysis)

**Our response**: Yes, this is logical indeed. We’ve revised that part.

**Comment**: 3. Results: the description of the table should be written above the table and cited in the manuscript. See journal criteria for table presentation, in the mean while in the result part write only the main findings, rather presenting all the findings from the table.

**Our response**: Thanks for drawing our attention to this. We’ve rearranged the tables.

**Comment**: 4. Conclusion: The conclusion in the abstract and in the manuscript is similar. This section should not repeat what has been mentioned in the abstract; rather, the research and policy importance obtained from the finding should be pointed out.

**Our response**: We agree with you. We’ve rewritten the conclusion section.

**Comment**: Furthermore, significant text overlap in method and discussion part seen in your manuscript E.g. https://www.researchgate.net/publication/303948025_HEALTHQUAL_a_multi-item_scale_for_assessing_healthcare_service_quality, www.researchgate.net › profile › Nestor-AsiamahThe Influence of Physicians’ Physical Activity Prescription , https://www.researchgate.net/publication/342601951_The_Influence_of_Physicians%27_Physical_Activity_Prescription_on_Indicators_of_Health_Service_Quality/ and www.ncbi.nlm.nih.gov › pmc › articlesA Randomised Controlled Trial of Triple Antiplatelet Therapy etc. I would recommend that the authors carefully re-check these.

**Our response**: This is an important observation. We’ve revised parts of the methodology and discussion to reduce the similarity.

**Reviewer #2:**

**Comment**: I am an academic teacher and for years has been preparing nursing staff to work with patients.I am convinced that the nursing staff is prepared for the programphysical activity counselling.Your article confirms this belief.You rightly emphasize the role of p[atient loyality. The research methodology is correct and the conclusions are well documented.I believe that the article is important and necessary, I have no reservations about publishing it.

**Our response**: We are indeed grateful for your time and priceless contribution to this manuscript.
